# Safety and efficacy of dexmedetomidine for long-term sedation in critically ill patients

**DOI:** 10.1007/s00540-013-1678-5

**Published:** 2013-08-03

**Authors:** Makoto Ozaki, Junzo Takeda, Keiji Tanaka, Yasuhiro Shiokawa, Shinichi Nishi, Kenichi Matsuda, Matsuyuki Doi, Yasuyuki Kakihana, Yuji Fujino, Masanori Takinami, Misa Kawai

**Affiliations:** 1 Department of Anesthesiology, Tokyo Women’s Medical University Hospital, Tokyo, Japan; 2 Department of Anesthesiology, School of Medicine, Keio University, Tokyo, Japan; 3 Division of CCU and ICU, Nippon Medical School Hospital, Tokyo, Japan; 4 Division of Intensive Care, Kinki University Hospital, Osaka, Japan; 5 Division of Intensive Care Medicine, Hyogo College of Medicine, Hyogo, Japan; 6 Department of Emergency and Critical Care Medicine, University of Yamanashi School of Medicine, Yamanashi, Japan; 7 Intensive Care Unit, Hamamatsu University School of Medicine, University Hospital, Shizuoka, Japan; 8 Intensive Care Unit, Kagoshima University Medical and Dental Hospital, Kagoshima, Japan; 9 Intensive Care Unit, Osaka University Hospital, Osaka, Japan; 10 Intensive Care Unit, The Jikei University School of Medicine, University Hospital, Tokyo, Japan; 11 Medical Affairs, Hospira Japan Co., Ltd, Osaka, Japan

**Keywords:** Dexmedetomidine, Long term, Sedation, Intensive care unit, Withdrawal

## Abstract

**Purpose:**

We evaluated the safety and efficacy of long-term administration of dexmedetomidine in patients in the intensive care unit (ICU). Primary endpoint was the incidence of hypotension, hypertension, and bradycardia. Secondary endpoints were withdrawal symptoms, rebound effects, the duration of sedation with Richmond Agitation-Sedation Scale (RASS) ≤ 0 relative to the total infusion time of dexmedetomidine, and the dose of additional sedatives or analgesics.

**Methods:**

Dexmedetomidine 0.2–0.7 μg/kg/h was continuously infused for maintaining RASS ≤ 0 in patients requiring sedation in the ICU. Safety and efficacy of short-term (≤24 h) and long-term (>24 h) dexmedetomidine administration were compared.

**Results:**

Seventy-five surgical and medical ICU patients were administered dexmedetomidine. The incidence of hypotension, hypertension, and bradycardia that occurred after 24 h (long-term) was not significantly different from that occurring within 24 h (short-term) (*P* = 0.546, 0.513, and 0.486, respectively). Regarding withdrawal symptoms, one event each of hypertension and headache occurred after the end of infusion, but both were mild in severity. Increases of mean arterial blood pressure and heart rate after terminating the infusion of dexmedetomidine were not associated with the increasing duration of its infusion. The ratio of duration with RASS ≤ 0 was ≥ 85 % until day 20, except day 9 (70 %) and day 10 (75 %). There was no increase in the dose of additional sedatives or analgesics after the first 24-h treatment period.

**Conclusions:**

Long-term safety of dexmedetomidine compared to its use for 24 h was confirmed. Dexmedetomidine was useful to maintain an adequate sedation level (RASS ≤ 0) during long-term infusion.

## Introduction

The importance of optimizing the levels of sedation in critical care has been increasingly recognized [1]. Many intensive care experts are focusing on maintaining a targeted “ideal” sedation level according to the individual patient’s condition to avoid adverse events such as prolonged mechanical ventilation, respiratory depression, pneumonia, delirium, psychological problems, and increased treatment costs resulting from oversedation [1–5]. However, optimizing the levels of sedation in intensive care unit (ICU) patients has been challenging, particularly in those requiring long-term sedation, frequently accompanied with severe conditions, and difficult to manage [6]. Although propofol and midazolam have been commonly used for long-time sedation, oversedation and respiratory depression have been regarded as unavoidable complications [4, 5, 7].

Dexmedetomidine is a selective alpha-2-adrenoceptor agonist. It exerts both sedative and analgesic effects via mechanisms different from other sedatives such as midazolam and propofol, and provides sedation characterized by prompt response to stimuli with no respiratory depression [8–11]. Although there have been several reports showing the effects of dexmedetomidine on long-term sedation [12–14], no prospective study has compared the safety and efficacy of short-term (within 24 h) and long-term (longer than 24 h) administration of dexmedetomidine for sedation in the ICU. We performed a prospective, multicenter trial to investigate the safety and efficacy of dexmedetomidine for long-term sedation in surgical and medical ICU patients.

## Materials and methods

### Study design

This was a prospective, single-arm, open-label, multicenter, phase III clinical study conducted at ten investigational sites in Japan between October 2007 and June 2008, aimed to obtain an approval for the long-term use (>24 h) of dexmedetomidine in Japan. It was approved by the Institutional Review Board at each site, and all the patients or legally acceptable representatives provided their written informed consent before enrollment. The study was conducted according to the Japanese Pharmaceutical Affairs Law, Japanese Good Clinical Practice, and relevant regulatory standards, and has been registered in ClinicalTrials.gov (NCT00526760) before recruitment of the first subject.

### Subjects

Inclusion criteria were patients admitted in either surgical or medical ICU aged ≥20 years, requiring mechanical ventilation and estimated duration of sedation >24 h, with American Society of Anesthesiologists physical status I to III (only those in surgical ICU). Exclusion criteria were patients with serious trauma in the central nervous system, terminal illness with life expectancy ≤30 days, with bleeding probably requiring surgical hemostasis, drug overdose within the last 30 days before study entry, pregnancy/lactation, contraindication to alpha-2-adrenoceptor agonists or antagonists, or difficulty in data collection or completing the study protocol. Patients required neuromuscular blocking agents except for tracheal intubation, received alpha-2-adrenoceptor agonists or antagonists within the last 30 days before participation in the study, patients who had participated in a trial with any experimental drug within 30 days before their admission into the ICU, or patients who had any symptom or factor that might increase the risk to the patients by participating in the study were also excluded.

### Treatment

Decision to start and terminate the infusion of dexmedetomidine was made by the investigators or sub-investigators. It was administered at 0.2–0.7 μg/kg/h for maintaining the sedation levels with Richmond Agitation-Sedation Scale (RASS) ≤ 0 [15]. Maximum duration of infusion was 28 days, and restarting infusion after termination was allowed within this limit. It was not necessary to discontinue the administration of the other sedatives or analgesics before starting infusion of dexmedetomidine. If necessary, additional sedatives and analgesics were given after assessing RASS or pain, respectively. Pain was assessed by direct communication with the patients or by an observation of clinical symptoms such as sweating, tachycardia, or hypertension. A 24-h observation period followed the dose administration. The patients were also followed for serious adverse events for 30 days after the end of the infusion.

### Efficacy and safety evaluation

The primary endpoint was the incidence rates of treatment-related hypotension, hypertension, and bradycardia, defined in the protocol as (1) hypotension: systolic blood pressure (SBP) < 60 mmHg, diastolic blood pressure (DBP) < 40 mmHg or decrease of SBP by ≥ 50 % from the baseline, requiring infusion or increase of the dose of vasopressors or fluid infusion ≥ 500 ml within 1 h; (2) hypertension: SBP > 180 mmHg, DBP > 100 mmHg, or increase of SBP by ≥ 50 % from the baseline, requiring infusion or increasing the dose of antihypertensive agents; and (3) bradycardia: heart rate (HR) < 40 bpm or decrease by ≥ 50 % from the baseline, requiring infusion or increase the dose of positive chronotropic medications or the use of a pacemaker. Treatment-related adverse events were defined as all the adverse events except those that were deemed “not related” to dexmedetomidine.

Secondary safety assessments included adverse events, withdrawal assessments of the incidence rates of withdrawal symptom-related adverse events (including increased blood pressure, tachycardia, nausea/vomiting, headache, tremor, anxiety, sweating, or agitation), and rebound assessments of the post-infusion changes in mean arterial blood pressure (MBP), HR, and rate-pressure product (RPP). If clinically important abnormal values were observed in hematology and blood chemistry, they were to be reported as adverse events. As with the primary endpoint, treatment-related adverse events were defined as all of the adverse events except that were deemed “not related” to dexmedetomidine.

Secondary efficacy endpoints included the ratio of duration with RASS ≤ 0 to the total duration of infusion of dexmedetomidine, and the dosage of additional sedatives and analgesics. Dexmedetomidine characteristic sedation level corresponds to a RASS of 0 to −2. However, this was a long-term study in ICU patients with a critical condition who would sometimes require deep sedation (RASS < −2), and the target sedation level during the infusion was set as RASS < 0.

### Statistical methods

Sample size was determined to detect at least one incidence of treatment-related hypotension, hypertension, or bradycardia. Assuming the incidence of bradycardia to be 5 %, the lowest among those events, 59 patients would be required to detect at least one incidence of 5 % treatment-related adverse events with a 95 % probability. Taking into account 20 % of the dropout cases, 80 patients were estimated as the sample size. It was also planned that approximately 15 % of the medical ICU patients would be enrolled.

For the primary safety analysis, the incidence rates per person per day of treatment-related adverse events including protocol-defined hypotension, hypertension, and bradycardia were calculated by dividing the number of those events by the sum of days of treatment for all patients including the 24-h observation period. The incidence rate during the first 24 h was compared with that after 24 h using the Sumi and Tango method of the score test [16]. In the secondary analysis, the incidence rates of the other treatment-related adverse events were analyzed as described for primary analysis. Descriptive statistics were used in the other assessments. The analysis was on the basis of the full analysis set of patients, which was defined as all the patients who received dexmedetomidine treatment. The level of significance in all statistical analysis was set at *α* = 0.05 (two-tailed).

## Results

### Patient demographics

A full analysis set consisted of 75 patients who received dexmedetomidine. Totally, 5 patients dropped out before dexmedetomidine treatment because of change in surgery date, persistent hemorrhage after surgery, or withdrawal of consent, and were excluded from the full analysis set. Of 75 patients, 52 (69.3 %) were surgical ICU and 23 (30.7 %) were medical ICU patients, respectively (Table 1). The medical ICU patients required a longer period of sedation compared to the surgical ICU patients. Maximum duration of dexmedetomidine treatment in the surgical ICU patients and the medical ICU patients was 5.6 and 19.9 days, respectively. Sixty-one of the 75 patients (81.3 %) received dexmedetomidine treatment both before and after extubation. Two of the 52 surgical ICU patients discontinued dexmedetomidine infusion during the first 24 h because of bradycardia or postoperative bleeding (Table 2).

**Table 1:** Baseline characteristics

Parameter	Surgical ICU, *n* (%)	Medical ICU, *n* (%)	Total, *n* (%)
	52 (69.3)	23 (30.7)	75 (100)
Age (years)			
Mean ± SD	66.4 ± 11.3	68.9 ± 12.9	67.1 ± 11.8
<65	17 (32.7)	7 (30.4)	24 (32.0)
≥65	35 (67.3)	16 (69.6)	51 (68.0)
Sex			
Male	37 (71.2)	18 (78.3)	55 (73.3)
Female	15 (28.8)	5 (21.7)	20 (26.7)
Body weight (kg)			
*n*	52	20	72
Mean ± SD	60.73 ± 11.57	57.20 ± 9.97	59.75 ± 11.19
Main surgical procedure			
Stent grafting	2 (3.8)		2 (3.8)
Patch closure	1 (1.9)		1 (1.9)
Bentall procedure	3 (5.8)		3 (5.8)
Coronary artery bypass graft	14 (26.9)		14 (26.9)
Subtotal esophagectomy	1 (1.9)		1 (1.9)
Blood vessel prosthesis implantation	13 (25.0)		13 (25.0)
Oropharynx tumor resection with neck dissection	1 (1.9)		1 (1.9)
Aneurysmectomy	2 (3.8)		2 (3.8)
Myxomectomy	1 (1.9)		1 (1.9)
Valve replacement/valvuloplasty	14 (26.9)		14 (26.9)
Specific medical disease			
Respiratory disease		8 (34.8)	8 (34.8)
Cardiac disease		8 (34.8)	8 (34.8)
Vascular disease		2 (8.7)	2 (8.7)
Other		5 (21.7)	5 (21.7)
Duration of surgery (h)			
<3	1 (1.9)		1 (1.9)
≥3, <5	17 (32.7)		17 (32.7)
≥5	34 (65.4)		34 (65.4)
ASA physical status			
I	0 (0.0)		0 (0.0)
II	25 (48.1)		25 (48.1)
III	27 (51.9)		27 (51.9)
History of smoking			
Non-smokers	24 (46.2)	8 (34.8)	32 (42.7)
Current smokers	6 (11.5)	6 (26.1)	12 (16.0)
Ex-smokers	22 (42.3)	9 (39.1)	31 (41.3)
History of alcohol use			
Non-alcohol users	23 (44.2)	8 (34.8)	31 (41.3)
Alcohol users	19 (36.5)	12 (52.2)	31 (41.3)
Ex-alcohol users	10 (19.2)	3 (13.0)	13 (17.3)

*ASA* American Society of Anesthesiologists, *ICU* intensive care unit

**Table 2:** Duration of treatment

Parameter (days)	Surgical ICU *n* (%)			Medical ICU *n* (%)			Total *n* (%)		
	Total	Before extubation	After extubation	Total	Before extubation	After extubation	Total	Before extubation	After extubation
	52 (69.3)	52 (69.3)	50 (66.7)	23 (30.7)	23 (30.7)	11 (14.7)	75 (100)	75 (100)	61 (81.3)
Mean ± SD	2.1 ± 1.2	0.9 ± 0.8	1.2 ± 0.9	7.4 ± 5.7	6.2 ± 5.8	2.4 ± 3.6	3.7 ± 4.1	2.5 ± 4.1	1.5 ± 1.8
Median	1.6	0.7	0.9	6.0	3.9	1.9	2.0	0.8	0.9
Q1–Q3	1.1–2.7	0.3–0.8	0.7–1.8	2.5–11.8	1.9–11.4	0.1–2.1	1.4–3.9	0.5–2.7	0.7–1.9
Min to max	0.1–5.6	0.1–3.2	0.01–3.8	1.0–19.9	0.6–19.9	0.1–12.8	0.1–19.9	0.1–19.9	0.01–12.8

*Q1* quartile 1, *Q3* quartile 3

### Safety

There were no differences in the incidence rates of treatment-related hypotension, hypertension, or bradycardia defined in the protocol, expressed as per person per day between the first 24 h and after 24 h (Table 3). There were also no differences in those values between the surgical and medical ICU patients.

**Table 3:** Incidence of treatment-related adverse events defined in the protocol within and after 24 h

	Number of events (incidence rate^a^) within 24 h	Number of events (incidence rate^b^) after 24 h	*P* value in Score test
Protocol-defined hypotension			
Total	3 (0.0400)	6 (0.0217)	0.546
Surgical ICU	2 (0.0385)	4 (0.0375)	0.951
Medical ICU	1 (0.0435)	2 (0.0118)	0.193
Protocol-defined hypertension			
Total	3 (0.0400)	6 (0.0217)	0.513
Surgical ICU	3 (0.0577)	3 (0.0281)	0.303
Medical ICU	0 (0.0000)	3 (0.0177)	0.530
Protocol-defined bradycardia			
Total	1 (0.0133)	0 (0.0000)	0.486
Surgical ICU	1 (0.0192)	0 (0.0000)	0.486
Medical ICU	0 (0.0000)	0 (0.0000)	–
Total			
Total	7 (0.0933)	12 (0.0435)	0.299
Surgical ICU	6 (0.1154)	7 (0.0656)	0.352
Medical ICU	1 (0.0435)	5 (0.0295)	0.644

Decreased and increased blood pressure according to Medical Dictionary for Regulatory Activities/Japanese version 11.0 was classified as hypotension and hypertension, respectively: *n* = 75 (Total), *n* = 52 (Surgical ICU), *n* = 23 (Medical ICU) within 24 h; *n* = 73 (Total), *n* = 50 (Surgical ICU), *n* = 23 (Medical ICU) after 24 h

^a^Incidence rate = number of events/person-days (person-days: 75 in total, 52 in surgical ICU, 23 in medical ICU)

^b^Incidence rate = number of events/person-days (person-days: 276 in total, 107 in surgical ICU, 169 in medical ICU)

The total incidence rate of treatment-related adverse events expressed as per person per day that occurred within 24 h was significantly higher than that after 24 h (Table 4). There were no differences in the incidence of each treatment-related adverse event within and after 24 h, with the exception that the incidence of increased blood pressure was higher within 24 h compared with that after 24 h. No treatment-related respiratory depression occurred. Three of 75 patients (4.0 %) developed delirium. One of three events was deemed as probably not related to dexmedetomidine, and the patient recovered 6 days after the onset of symptoms. Another two events were deemed as not related to dexmedetomidine, and the patients recovered about 5 h and 9 days after the onset of symptoms, respectively. Seven patients had died after the end of the dexmedetomidine infusion of respiratory failure, cardiac failure, pneumonia aspiration, multiorgan failure, or sepsis. These events were not considered related to dexmedetomidine infusion, and no other serious adverse events related to dexmedetomidine infusion were observed.

**Table 4:** Incidence of treatment-related adverse events within and after 24 h

Treatment-related adverse events	Number of events (incidence rate) within 24 h (*n* = 75)	Number of events (incidence rate) after 24 h (*n* = 73)	*P* value in score test
Total	18 (0.2400)	27 (0.0978)	0.014
Decreased blood pressure	5 (0.0667)	13 (0.0471)	0.442
Increased blood pressure	9 (0.1200)	7 (0.0254)	0.019
Bradycardia	2 (0.0267)	1 (0.0036)	0.558
Platelet count decreased	1 (0.0133)	0 (0.0000)	0.061
Hepatic function abnormal	1 (0.0133)	0 (0.0000)	0.061
Hypotension	0 (0.0000)	3 (0.0109)	0.681
Eosinophilia	0 (0.0000)	1 (0.0036)	0.767
Delirium	0 (0.0000)	1 (0.0036)	0.625
Headache	0 (0.0000)	1 (0.0036)	0.540

Decreased blood pressure and hypotension were separately counted following Medical Dictionary for Regulatory Activities/Japanese version 11.0: incidence rate = number of events/person-days (person-days: 75 within 24 h, 276 after 24 h)

**Fig. 1:**
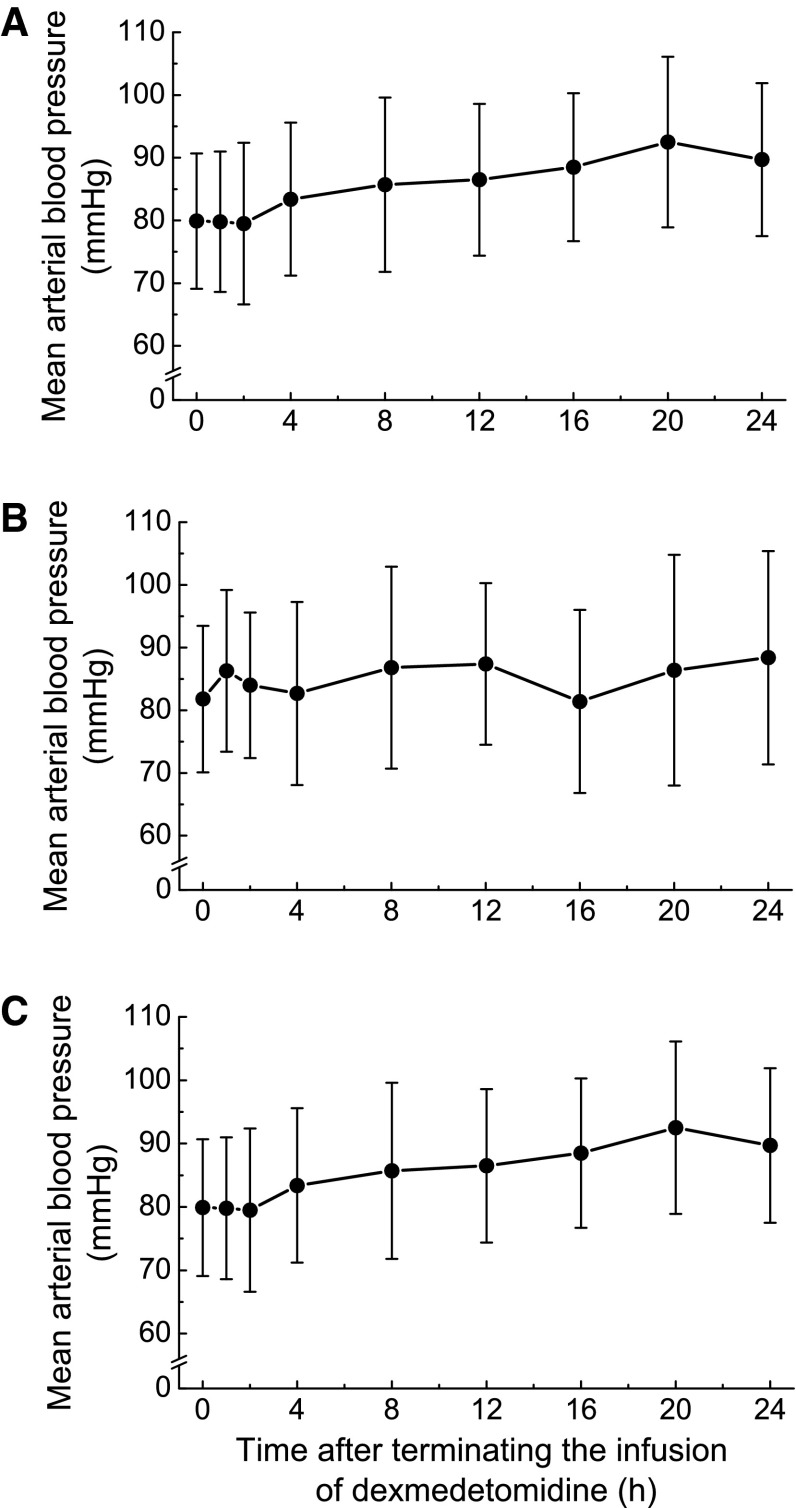
Mean arterial blood pressure after terminating infusion of dexmedetomidine in patients receiving dexmedetomidine for ≤2 days (*n* = 38) (**a**), 3–5 days (*n* = 24) (**b**), or >5 days (*n* = 13) (**c**). Values are expressed as mean ± SD of 37 or 38 (**a**), 24 (**b**), and 12 or 13 (**c**) individuals

**Fig. 2:**
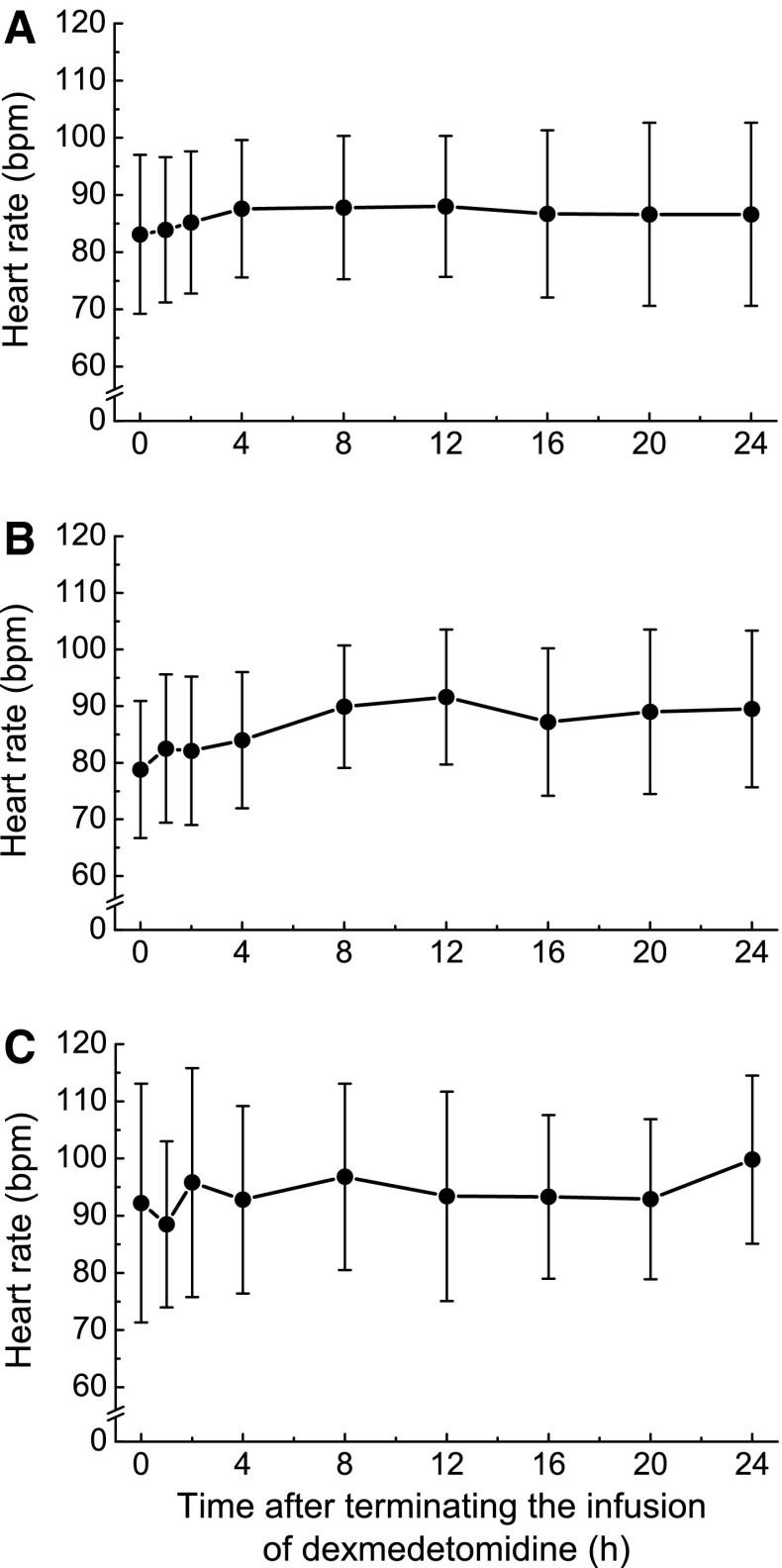
Heart rate after terminating infusion of dexmedetomidine in patients receiving dexmedetomidine for ≤2 days (*n* = 38) (**a**), 3–5 days (*n* = 24) (**b**), or >5 days (*n* = 13) (**c**). Values are expressed as mean ± SD of 37 or 38 (**a**), 24 (**b**), and 12 or 13 (**c**) individuals

**Fig. 3:**
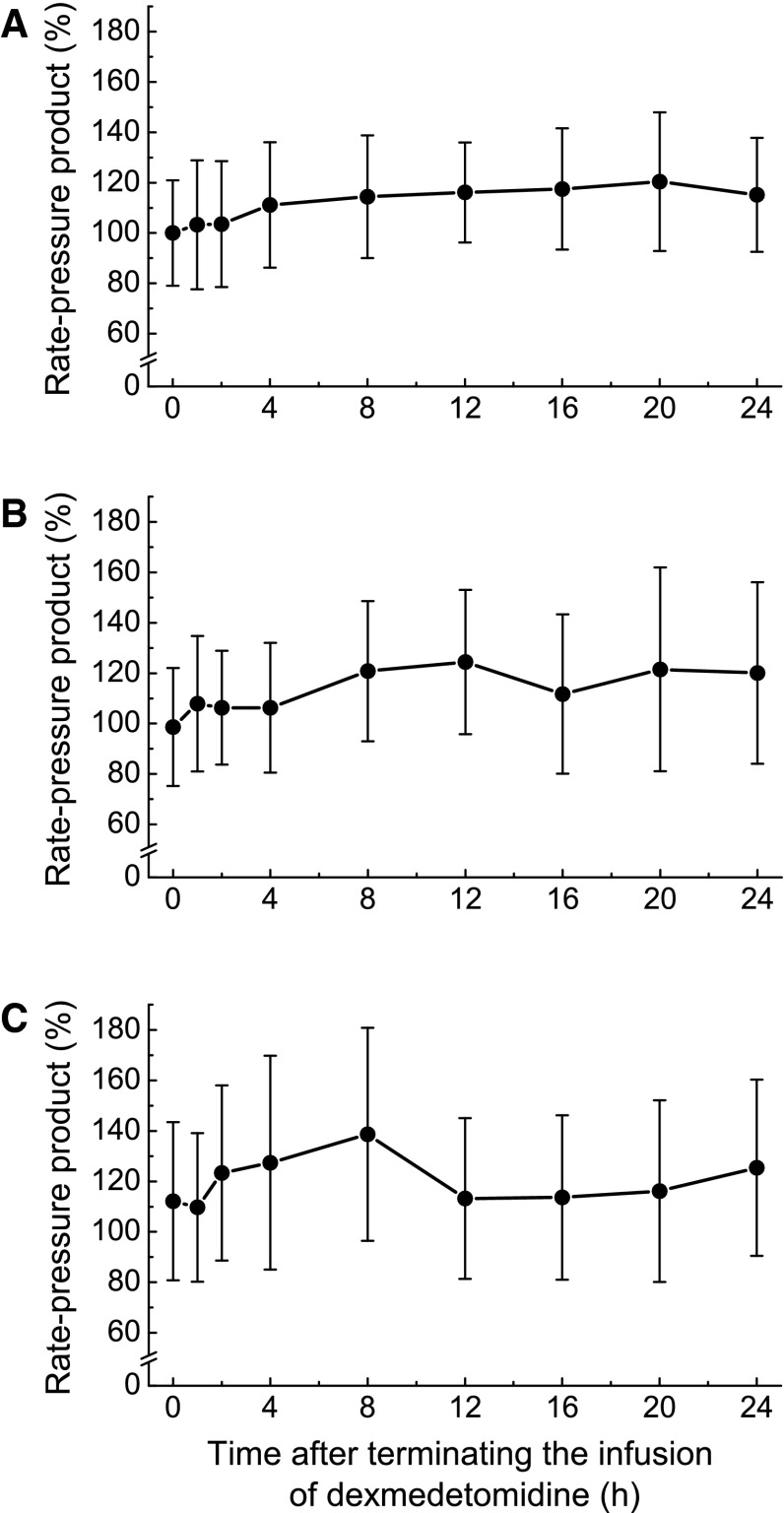
Rate–pressure product after terminating infusion of dexmedetomidine in patients receiving dexmedetomidine for ≤2 days (*n* = 38) (**a**), 3–5 days (*n* = 24) (**b**), or >5 days (*n* = 13) (**c**). Values are expressed as mean ± SD of 37 or 38 (**a**), 24 (**b**), and 12 or 13 (**c**) individuals

A total of 13 adverse events related to withdrawal symptoms were observed in 9 of 75 patients, and all the adverse events were mild with the exception of 1 moderate headache event (Table 5). One event each of increased blood pressure and headache were considered treatment related, and each event was mild in severity. MBP, HR, and RPP modestly increased after the termination of long-term infusion of dexmedetomidine. Changes were not associated with the increasing duration of dexmedetomidine infusion (Figs. 1, 2, 3).

**Table 5:** Adverse events related to withdrawal symptoms

Adverse events	Total		Not related to treatment		Related to treatment	
	No. of events	No. of patients with events (%)	No. of events	No. of patients with events (%)	No. of events	No. of patients with events (%)
Total	13	9 (12.0)	11	8 (10.7)	2	2 (2.7)
Increased blood pressure	7	6 (8.0)	6	6 (8.0)	1	1 (1.3)
Tachycardia	2	2 (2.7)	2	2 (2.7)	0	0 (0.0)
Nausea/vomiting	2	2 (2.7)	2	2 (2.7)	0	0 (0.0)
Headache	2	2 (2.7)	1	1 (1.3)	1	1 (1.3)
Tremor	0	0 (0.0)	0	0 (0.0)	0	0 (0.0)
Anxiety	0	0 (0.0)	0	0 (0.0)	0	0 (0.0)
Sweating	0	0 (0.0)	0	0 (0.0)	0	0 (0.0)
Agitation	0	0 (0.0)	0	0 (0.0)	0	0 (0.0)

*n* = 75

### Efficacy

**Fig. 4:**
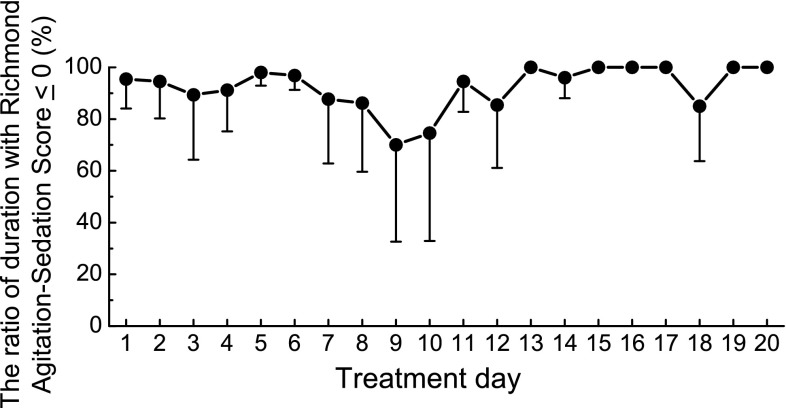
The ratio of duration with the Richmond Agitation-Sedation Scale ≤ 0 during dexmedetomidine treatment was calculated for each patient and mean ± SD values were analyzed. There were 75 patients on day 1, 73 on day 2, 37 on day 3, 26 on day 4, 18 on day 5, 13 on day 6, 11 on day 7, 9 on day 8, 8 on days 9–12, 5 on day 13, 4 on days 14–15, 2 on days 16–18, and 1 on days 19–20

During administration of the study drug, the patients were within the target sedation range (RASS ≤ 0) 85 % of the time, except on days 9–10. On days 9–10, a medical ICU patient with agitation (including tube pulling and aggressive behavior) and another patient with daytime arousal (RASS > 0) were observed, and the ratio of duration in the target sedation range decreased to approximately 70–75 % (Fig. 4).

The number of patients who required additional sedatives or analgesics did not increase after 24 h compared to the first 24 h (Table 6). Forty of 75 patients (53.3 %) and 24 of 73 patients (32.9 %) required additional sedatives during the first 24 h and after 24 h, respectively, and 21 of 75 patients (28.0 %) and 19 of 73 patients (26.0 %) required additional analgesics during the first 24 h and after 24 h, respectively.

**Table 6:** Number of patients who required additional sedatives or analgesics

Agent	*n* (%) within 24 h (*n* = 75)	*n* (%) after 24 h (*n* = 73)
Additional sedatives	40 (53.3)	24 (32.9)
Propofol		
IVB	13 (17.3)	13 (17.8)
IVC	29 (38.7)	21 (28.8)
Midazolam		
IVB	7 (9.3)	4 (5.5)
IVC	3 (4.0)	4 (5.5)
Fentanyl (administered as a sedative)		
IVB	3 (4.0)	4 (5.5)
IVC	3 (4.0)	4 (5.5)
Haloperidol		
IVB	0	1 (1.4)
IVC	1 (1.3)	1 (1.4)
Additional analgesics	21 (28.0)	19 (26.0)
Fentanyl		
IVB	5 (6.7)	7 (9.6)
IVC	8 (10.7)	8 (11.0)
Buprenorphine		
IVB	5 (6.7)	2 (2.7)
IVC	2 (2.7)	1 (1.4)
REC	1 (1.3)	1 (1.4)
Pentazocine		
IVB	4 (5.3)	0
IM	0	1 (1.4)
Diclofenac		
REC	1 (1.3)	2 (2.7)
Droperidol		
ED	1 (1.3)	1 (1.4)
Flurbiprofen		
IVB	1 (1.3)	1 (1.4)
IVC	1 (1.3)	0
Loxoprofen		
PO	2 (2.7)	1 (1.4)
Morphine		
IVB	0	1 (1.4)
IVC	0	3 (4.1)
ED	1 (1.3)	1 (1.4)
Remifentanil		
IVC	1 (1.3)	0
Ropivacaine		
SC	1 (1.3)	0
ED	1 (1.3)	1 (1.4)

*ED* epidural administration, *IVB* intravenous bolus injection, *IVC* continuous intravenous infusion, *IM* intramuscular administration, *PO* oral administration, *REC* rectal administration, *SC* subcutaneous administration

There was no increase in the dose of additional sedatives or analgesics after 24 h administration (Tables 7, 8). Propofol and midazolam were administered to many patients as additional sedatives. Although neither fentanyl nor haloperidol is a sedative, some patients were administered these drugs for sedation. Fentanyl, buprenorphine, pentazocine, or other analgesics were administered for analgesia.

**Table 7:** Dose of additional sedatives

	Route	Day 1	Day 2	Day 3	Day 4	Day 5	Day 6	Day 7	Day 8
		(*n* = 75)	(*n* = 73)	(*n* = 37)	(*n* = 26)	(*n* = 18)	(*n* = 13)	(*n* = 11)	(*n* = 9)
Propofol	IVB	*n* = 13	*n* = 8	*n* = 4	*n* = 4	*n* = 3	*n* = 3	*n* = 1	*n* = 4
		60.4 ± 52.4	60.0 ± 30.7	36.3 ± 32.5	32.5 ± 15.0	43.3 ± 15.3	46.7 ± 15.3	40.0	70.0 ± 74.4
	IVC	*n* = 29	*n* = 16	*n* = 10	*n* = 8	*n* = 5	*n* = 4	*n* = 2	*n* = 3
		777.1 ± 1,038.0	1,338.3 ± 1,535.4	874.8 ± 540.4	951.3 ± 854.1	858.8 ± 904.2	753.9 ± 1,040.9	2,233.8 ± 913.9	838.9 ± 390.5
Midazolam	IVB	*n* = 7	*n* = 3	*n* = 1	*n* = 1	*n* = 1	*n* = 2	*n* = 1	*n* = 1
		7.1 ± 4.8	2.2 ± 2.4	15.0	10.0	0.6	5.3 ± 6.6	5.0	3.0
	IVC	*n* = 3	*n* = 3	*n* = 2	*n* = 3	*n* = 2	*n* = 1	*n* = 2	*n* = 3
		133.7 ± 183.6	77.5 ± 115.3	129.8 ± 155.9	107.3 ± 154.5	181.8 ± 252.1	140.0	77.0 ± 53.7	22.9 ± 22.3
Fentanyl	IVB	*n* = 3	*n* = 4	*n* = 1	*n* = 1	*n* = 1	*n* = 1	*n* = 1	–
		0.15 ± 0.22	0.45 ± 0.77	0.25	0.20	0.40	0.125	0.03	
	IVC	*n* = 3	*n* = 3	*n* = 2	*n* = 3	–	–	*n* = 1	*n* = 2
		0.42 ± 0.28	1.39 ± 2.22	2.34 ± 3.04	1.84 ± 2.96			0.65	0.46 ± 0.65
Haloperidol	IVC	*n* = 1	*n* = 1	–	–	–	–	–	–
		5.0	5.0						

	Route	Day 9	Day 10	Day 11	Day 12	Day 13	Day 14	Day 15	Day 16
		(*n* = 8)	(*n* = 8)	(*n* = 8)	(*n* = 8)	(*n* = 5)	(*n* = 4)	(*n* = 4)	(*n* = 2)
Propofol	IVB	*n* = 2	–	*n* = 2	–	*n* = 1	*n* = 1	–	–
		15.0 ± 7.1		50.0 ± 14.1		30.0	40.0		
	IVC	*n* = 1	*n* = 1	*n* = 3	*n* = 1	*n* = 1	*n* = 1	–	–
		983.3	4,817.5	1,181.1 ± 1,439.3	127.5	60.5	239.0		
Midazolam	IVB	*n* = 1	*n* = 1	–	–	–	–	–	–
		2.1	2.5						
	IVC	*n* = 2	*n* = 2	*n* = 2	*n* = 2	*n* = 1	*n* = 1	–	–
		38.1 ± 38.1	54.4 ± 50.3	6.8 ± 5.9	57.0 ± 55.2	96.0	14.6		
Fentanyl	IVB	*n* = 1	–	–	–	–	–	–	*n* = 1
		0.30							0.15
	IVC	–	–	*n* = 1	*n* = 1	*n* = 1	*n* = 1	–	–
				0.11	0.96	0.96	0.15		
Haloperidol	IVB	*n* = 1	–	–	–	–	–	–	–
		10.0							
	IVC	–	–	–	–	–	–	–	–

	Route	Day 17	Day 18	Day 19	Day 20				
		(*n* = 2)	(*n* = 2)	(*n* = 1)	(*n* = 1)				
–	–	–	–	–	–				

Values are expressed as mean ± SD (mg)

*IVB* intravenous bolus injection, *IVC* continuous intravenous infusion

**Table 8:** Dose of additional analgesics

	Route	Day1	Day2	Day3	Day4	Day5	Day6	Day7	Day8
		(*n* = 75)	(*n* = 73)	(*n* = 37)	(*n* = 26)	(*n* = 18)	(*n* = 13)	(*n* = 11)	(*n* = 9)
Fentanyl	IVB	*n* = 5	*n* = 4	*n* = 1	*n* = 1	–	*n* = 1	*n* = 1	–
		0.13 ± 0.10	0.06 ± 0.03	0.03	0.01		0.03	0.01	
	IVC	*n* = 8	*n* = 7	*n* = 2	*n* = 3	*n* = 2	*n* = 2	–	*n* = 1
		0.40 ± 0.30	0.28 ± 0.23	0.41 ± 0.02	0.34 ± 0.29	0.49 ± 0.03	0.57 ± 0.56		0.35
Buprenorphine	IVB	*n* = 5	–	–	–	*n* = 1	–	–	*n* = 1
		0.17 ± 0.11				0.20			0.02
	IVC	*n* = 2	*n* = 1	*n* = 1	*n* = 1	*n* = 1	*n* = 1	*n* = 1	*n* = 1
		0.23 ± 0.05	0.40	0.40	0.40	0.40	0.40	0.40	0.28
	REC	*n* = 1	*n* = 1	–	–	–	–	–	–
		0.20	0.20						
Pentazocine	IVB	*n* = 4	–	–	–	–	–	–	–
		15.0 ± 0.0							
	IM	–	–	–	*n* = 1	–	–	–	–
					15.0				
Diclofenac	REC	*n* = 1	*n* = 2	–	–	–	–	–	–
		25.0	18.8 ± 8.8						
Droperidol	ED	*n* = 1	*n* = 1	–	–	–	–	–	–
		0.42	1.99						
Flurbiprofen	IVB	*n* = 1	–	–	–	–	–	–	*n* = 1
		50.0							50.0
	IVC	*n* = 1	–	–	–	–	–	–	–
		50.0							
Loxoprofen	PO	*n* = 2	–	*n* = 1	–	–	–	–	–
		60.0 ± 0.0		120.0					
Morphine	IVB	–	–	–	–	–	*n* = 1	–	–
							1.0		
	IVC	–	*n* = 2	–	–	–	*n* = 1	–	–
			5.6 ± 1.6				7.0		
	ED	*n* = 1	*n* = 1	–	–	–	–	–	–
		0.7	3.2						
Remifentanil	IVC	*n* = 1	–	–	–	–	–	–	–
		1.10							
Ropivacaine	SC	*n* = 1	–	–	–	–	–	–	–
		7.5							
	ED	*n* = 1	*n* = 1	–	–	–	–	–	–
		50.0	238.3						

	Route	Day9	Day10	Day11	Day12	Day13	Day14	Day15	Day16
		(*n* = 8)	(*n* = 8)	(*n* = 8)	(*n* = 8)	(*n* = 5)	(*n* = 4)	(*n* = 4)	(*n* = 2)
Fentanyl	IVB	–	–	*n* = 1	–	–	–	–	–
				0.10					
	IVC	*n* = 1	*n* = 1	*n* = 1	*n* = 1	*n* = 1	*n* = 1	*n* = 1	*n* = 1
		0.04	0.60	0.48	0.30	0.60	0.60	0.60	0.81
Buprenorphine	IVB	–	–	–	*n* = 1	–	–	–	–
					0.02				
	IVC	–	–	–	*n* = 1	*n* = 1	–	–	–
					0.35	0.03			

	Route	Day17	Day18	Day19	Day20				
		(*n* = 2)	(*n* = 2)	(*n* = 1)	(*n* = 1)				
Fentanyl	IVB	–	–	–	–				
	IVC	*n* = 1	*n* = 1	*n* = 1	–				
		0.96	0.96	1.05					

Values are expressed as mean ± SD (mg)

*ED* epidural administration, *IVB* intravenous bolus injection, *IVC* continuous intravenous infusion, *IM* intramuscular administration, *REC* rectal administration, *PO* oral administration, *SC* subcutaneous administration

## Discussion

The purpose of this study was to evaluate the safety and efficacy of dexmedetomidine for long-term use. We compared the safety and efficacy of dexmedetomidine during the initial period of ≤24 h and the subsequent period. This design and the approach in this prospective study were unique.

In contrast to other sedatives, dexmedetomidine is not associated with respiratory depression [10, 11] and can be administered continuously throughout intubation as well as after extubation. Dexmedetomidine provides a light to moderate level of sedation with the unique feature of arousability [9]. Propofol and midazolam are not typically used after extubation because of the effects of respiratory depression and potential to produce deeper sedation [17, 18]. Therefore, it was decided not to use either as a comparator. Using a placebo as a comparator was denied because of ethical considerations.

Patients who require long-term sedation are typically in more critical condition compared to patients who require short-term sedation, and they sometimes need deep sedation. When deep sedation is required in the usual ICU setting, other sedatives may be used alone or concomitantly with dexmedetomidine. Therefore, the concomitant use of other sedatives as in the usual ICU setting was allowed in this study. In this study, it was considered more important to conduct a long-term investigation according to its use in the usual ICU setting.

Long-term infusion of dexmedetomidine was well tolerated in both surgical and medical ICU patients. The results of this study showed no increase in treatment-related hypotension, hypertension, bradycardia, or other adverse events during a long-term administration period compared to the initial 24 h of treatment. Although MBP, HR, and RPP modestly increased after the termination of dexmedetomidine, the changes were not associated with the increasing duration of dexmedetomidine infusion. There was no evidence suggesting a withdrawal syndrome or rebound effect, which was a concern after the termination of long-term administration of an α2-receptor agonist. Tapering of the dexmedetomidine dose was not necessary, consistent with previous studies [13, 14]. The ratio of duration with RASS ≤ 0 to the total duration of infusion of dexmedetomidine did not decrease after 24 h. Furthermore, neither the number of patients who required additional sedatives/analgesics nor the dose of additional sedatives/analgesics increased over time.

Infusion of a loading dose is required to rapidly increase the plasma concentration of dexmedetomidine; however, it may be accompanied with adverse effects such as hypertension [8, 10]. Although loading infusion was an option for this study and was available at the investigator’s discretion, no patients had received a loading dose. In surgical ICU patients, study drug administration was initiated when the residual effect of anesthesia during surgery was observed. In medical ICU patients, study drug administration was initiated when the effects of other sedatives were still sufficient. While the patients were sedated, the other sedatives were switched to dexmedetomidine or dexmedetomidine were concomitantly administered with the other sedatives. Thus, no loading dose was necessary.

This study included two patients under noninvasive positive pressure ventilation (NPPV) in the medical ICU. Sedation with dexmedetomidine is desirable in these patients as those receiving NPPV should be conscious to minimize the risk of aspiration pneumonia from lack of airway protection [19–21]. On the other hand, these patients experience discomfort and may develop agitation during NPPV from the use of a face mask. In the present study, dexmedetomidine provided adequate sedation in patients receiving NPPV without any evidence of respiratory depression.

Although the post-extubation period was not the main focus in this study and there were no separate sub-analysis data for the post-extubation period only, the efficacy and safety evaluations included not only the intubation period but also the post-extubation period. Of the 75 patients, 61 (81.3 %) received dexmedetomidine after extubation. Long-term use of dexmedetomidine after extubation in these patients was effective, and no adverse event indicating respiratory depression was observed.

There have been several previous reports that dexmedetomidine reduces the incidence of delirium [13, 22]. In this study, 3 of 75 patients (4.0 %) developed delirium, 1 of which events was deemed as probably not related and the others as not related to dexmedetomidine. However, this study was not a comparative study, and there was no use of the Confusion Assessment Method for the ICU [23] to assess delirium. Therefore, we cannot report on the effects of dexmedetomidine on delirium.

Oversedation leads to poor patient prognosis and increased treatment costs [1–5]. Therefore, it is desirable to avoid oversedation and to maintain the patient at an ideal sedation level. Additionally, the ideal level of sedation differs for each patient because the condition of patients managed in ICU settings is highly variable. In this study, investigators used dexmedetomidine as a fundamental sedative to provide a light to moderate level of sedation (in which patients were easily arousable and cooperative). Other sedatives were concomitantly administered, not only when sedation management was difficult with dexmedetomidine alone but also when deep sedation was necessary. As a result, 61.6 % and 38.4 % of patients received additional sedatives and analgesics, respectively.

Although the interaction of concomitant sedatives needs to be carefully monitored, the concomitant use of other sedatives with dexmedetomidine provides benefits in long-term use, as it utilizes each of the sedative’s properties as needed. Other sedatives commonly used for long-term ICU sedation include midazolam and propofol. Midazolam has less vasodilatory effect compared to dexmedetomidine or propofol [24]. However, long-term use of midazolam demonstrates significant interindividual variation in pharmacokinetics and produces an active metabolite, which results in a prolonged recovery to consciousness after long-term treatment [25, 26]. In addition, a patient may also develop tolerance after long-term use of midazolam [24, 27]. Long-term use of propofol has a short elimination half-life and rapid offset to consciousness [25]. However, long-term use of propofol includes an increased risk of infection by the same route, a risk of excessive blood lipids associated with the lipid emulsion formulation, development of tolerance, and propofol infusion syndrome [7, 17, 27, 28]. Potential advantages of dexmedetomidine for long-term use include the arousability feature, and that it is not associated with respiratory depression, both of which can facilitate weaning and extubation. Dexmedetomidine also has the potential to reduce the incidence of delirium, which increases with prolonged ICU stay [13, 22]. The concomitant uses of other sedatives or analgesics were not increased over time, and the majority of patients were maintained at the target sedation levels without any increase in dose, suggesting that there was no development in tolerance. A potential disadvantage of dexmedetomidine is that it should be used very cautiously in patients with hypotension and/or bradycardia [14, 28].

## Conclusions

The long-term safety of dexmedetomidine compared to its use for 24 h was confirmed. Dexmedetomidine was useful to maintain adequate sedation levels (RASS ≤ 0) in both surgical and medical ICU patients during long-term infusion. No clinically significant withdrawal symptoms or rebound effects were observed after the end of long-term treatment. The ratio of duration with RASS ≤ 0 did not decrease after the first 24 h administration, and there was no increase in dose of additional sedatives or analgesics, suggesting no tolerance occurred. Considering its unique properties, investigators used dexmedetomidine as the fundamental sedative, and additional sedatives and analgesics were added based on each patient’s condition.
